# Poisson’s Ratio and Young’s Modulus of Lipid Bilayers in Different Phases

**DOI:** 10.3389/fbioe.2014.00008

**Published:** 2014-04-22

**Authors:** Tayebeh Jadidi, Hamid Seyyed-Allaei, M. Reza Rahimi Tabar, Alireza Mashaghi

**Affiliations:** ^1^Department of Physics, University of Osnabrück, Osnabrück, Germany; ^2^Department of Physics, Sharif University of Technology, Tehran, Iran; ^3^Institute of Physics, Carl-von-Ossietzky University, Oldenburg, Germany; ^4^Kavli Institute of Nanoscience, Delft University of Technology, Delft, Netherlands

**Keywords:** membrane mechanics, Young’s modulus, Poisson’s ratio, lipid bilayer, soft matter

## Abstract

A general computational method is introduced to estimate the Poisson’s ratio for membranes with small thickness. In this method, the Poisson’s ratio is calculated by utilizing a rescaling of inter-particle distances in one lateral direction under periodic boundary conditions. As an example for the coarse grained lipid model introduced by Lenz and Schmid, we calculate the Poisson’s ratio in the gel, fluid, and interdigitated phases. Having the Poisson’s ratio, enable us to obtain the Young’s modulus for the membranes in different phases. The approach may be applied to other membranes such as graphene and tethered membranes in order to predict the temperature dependence of its Poisson’s ratio and Young’s modulus.

## Introduction

Elastic properties play an important role in a number of membrane processes, as for example, membrane fusion (Chernomordik and Kozlov, 2008) and modulations of membrane channel activities (Schmidt and MacKinnon, 2008; Sansom and Biggin, 2010; Mashaghi et al., 2013b). In cells, the outer membranes are supported by the underlying actin networks. The mechanical stress is then dominated by the associated actin cytoskeleton at length scales larger than the mesh size of the actin network (30–300 nm) (Morone et al., 2006). On small length scales, however, the contribution of the lipid bilayer will dominates. As such, efforts have been made to study the mechanical properties of bilayer patches with dimensions close to the mesh size of the actin cytoskeleton (Claesson et al., 2011).

When considering a membrane as a two-dimensional body, i.e., neglecting its thickness, its mechanical properties in the absence of anisotropies can be characterized by two elastic constants according to continuum elasticity theory. In common practice of material characterization, these parameters are typically the Young’s modulus and the Poisson’s ratio. Estimates for the Young’s modulus of membranes have been provided by experiments (Tierney et al., 2005; Popescu et al., 2006). However, the measuring of the Poisson’s ratio is not straightforward, due to the small thickness of membranes in the nanometer range (Mitchell et al., 2003; Martins et al., 2009). From the theoretical aspect, mechanical properties of lipid membranes are commonly investigated based on the Helfrich Hamiltonian. The main physical quantity obtained from such studies is the bending rigidity. The Young’s modulus and Poisson’s ratio are interrelated by formula that incorporate the bending rigidity, but neither Young’s modulus nor Poisson’s ratio have been determined separately so far.

Simulations and theoretical models have been used to provide important information on elastic (Goetz and Lipowsky, 1998; Lindahl and Edholm, 2001; Ayton et al., 2002) and viscous properties of lipid bilayers (Jeon and Voth, 2005). Investigating the mechanical properties of thin films is not limited to biomembranes and represents an active area of research in materials science. Efforts have been put into predicting the Poisson’s ratio of films made of various materials by means of computer simulations. For instance Galvao et al. has employed molecular dynamics simulations using reactive empirical bond-order potentials to investigate the mechanical properties of graphene nanoribbons (Martins and Galvao, 2010). Baughman et al. proposed a model to estimate the Poisson’s ratio of fiber networks and successfully applied it to carbon nanotube sheets (buckypaper) (Hall et al., 2008).

In this work, we introduce a method for determining the Poisson’s ratio ν in simulations and apply it to the coarse grained lipid membrane model, which was introduced by Lenz and Schmid (2005). This method is general and applicable to any other surfaces. After determining the bending rigidity *k_c_* from the power spectrum of membrane height fluctuations, we are able to calculate the Young’s modulus *E*.

## Materials and Methods

**Figure 1:**
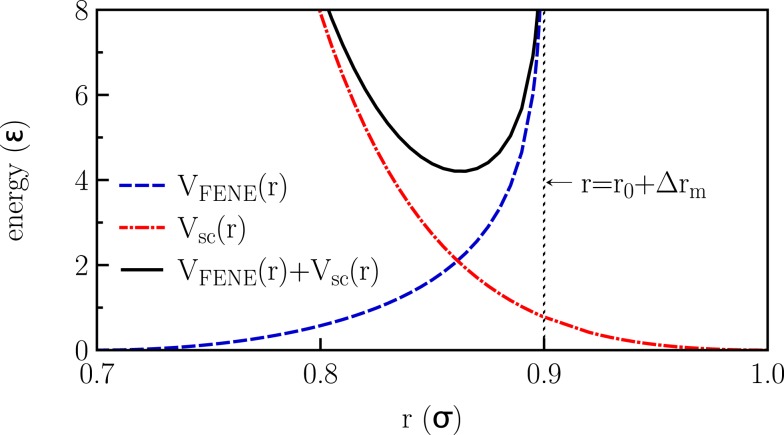
**Interactions applied in the model**.

Monte Carlo simulations of lipid bilayers with periodic boundary conditions in lateral directions were carried out for the coarse grained model introduced by Lenz and Schmid (2005). In this model, single-tail amphiphiles are considered, which are represented by six tail beads and one slightly larger head bead (with a size ratio of 1–1.1). Beads belonging to one molecule are connected via finitely extensible non-linear elastic (FENE) springs (Grest and Kremer, 1986) with a bond stretching potential Figure 1:

(1) $$V_{\text{FENE}}\;\left(r\right)=-\frac{\upsilon_{\text{FENE}}}{2}{\left(\Delta r_{m}\right)}^{2}\log\left[1-{\left(\frac{r-r_{0}}{\Delta r_{m}}\right)}^{2}\right]$$

Here *r* is the distance between adjacent beads, ν_FENE_ characterizes the strength of the spring, *r*_0_ is the optimal bond length (no stretching), and Δ*r_m_* is the maximal stretching distance. The stiffness of the tails is taken into account by a harmonic bond-angle potential

(2) $$V_{\text{ba}}\left(\theta_{ijk}\right)=\upsilon_{\text{ba}}\left(1-\cos\theta_{ijk}\right)$$

where θ*_ijk_* is the bond-angle associated with three adjacent beads. Both the non-bonded beads belonging to the same molecule and the beads belonging to different molecules interact via a soft-core potential:

(3a) $$\begin{array}{ll} V_{\text{sc}}\;\left(r\right) & =\left[V_{\text{LJ}}\left(r\right)-V_{\text{LJ}}\left(r_{\text{c}}\right)\right]\;\theta \left(r_{\text{c}}-r\right) \end{array}$$

(3b) $$\begin{array}{ll} V_{\text{LJ}}\;\left(r\right) & =\;\epsilon \left[{\left(\frac{\sigma_{\text{LJ}}}{r}\right)}^{12}-2{\left(\frac{\sigma_{\text{LJ}}}{r}\right)}^{6}\right] \end{array}$$

where θ_(.)_ is the Heaviside step function. The solvent molecules are represented by a phantom model of beads that interact with the lipid beads via *V*_SC_(*r*) (with same parameters as the head beads) but do not interact with themselves. All model parameters have been chosen according to the referenced model (Lenz and Schmid, 2005) and are summarized in Table 1.

**Table 1:** **Interaction potentials of the referenced model and corresponding parameters**.

Interaction type	Potential	Parameters
Tail–tail	*V_sc_*	ϵ = 1, σ_LJ_ = 1, *r_c_* = 2σ_LJ_
Head–tail		ϵ = 1, σ_LJ_ = 1.05, *r_c_* = 1σ_LJ_
Solvent–tail		ϵ = 1, σ_LJ_ = 1.1, *r_c_* = 1σ_LJ_
Head–head	
Solvent–head	
Solvent–solvent	None	
Bond length	*V*_FENE_	ν_FENE_ = 100, *r*_0_ = 0.7, Δ*r_m_* = 0.2
Bond angle	*V*_ba_	ν_ba_ = 4.7

**r* is the distance of the connecting vector of two particles and θ is the angle between every three adjacent beads. The SI values are calculated using σ_LJ_ ~ 6Å and ϵ ~ 3.6.10^−21^*J* (Neder et al., 2010)*.

Lipid bilayers exhibit a rich spectrum of structures and phase transitions (Nagle and Tristram-Nagle, 2000; Illya et al., 2005; Seto et al., 2008; Thakkar et al., 2011). The fluid state at high temperatures is characterized by a disordered arrangement of the lipid tails and a comparatively high lipid mobility. Upon cooling, this fluid state undergoes a phase transition to a gel state, where the lipid molecules are more ordered and have a lower mobility. Other possible phases are the interdigitated phase, in which lipid tails from opposing monolayers interpenetrate.

**Figure 2:**
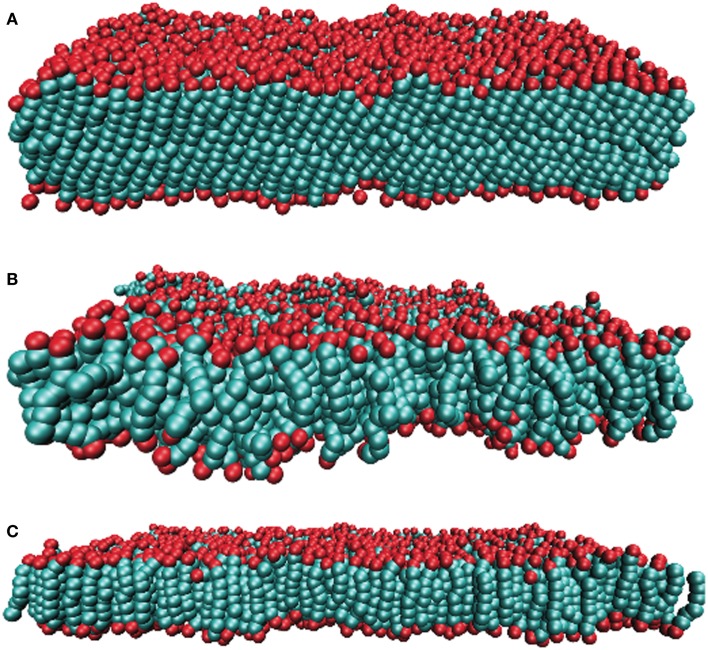
**Snapshots of the simulated lipid membrane in the (A) gel, (B) fluid, and (C) interdigitated phase**. Lipid’s head beads and tail bead are shown in red and green, respectively.

By scanning the phase diagram of the referenced model (Lenz, 2007), firstly we equilibrated lipid bilayers for about two millions Monte Carlo (MC) steps to produce different phases for the aim of this work, see Figure 2. In the reduced units, ϵ/k_B_ for the temperature T and $\epsilon /\sigma_{\text{LJ}}^{3}$ for the pressure *P*, the corresponding thermodynamic variables are: *P* = 2 and *T* = 1.08 for the gel phase, *P* = 1 and *T* = 1.3 for the fluid phase, *P* = 0.5 and *T* = 1.16 for the interdigitated phase. The characteristic parameters for different phases including the average chain length $\bar{l}$, thickness of the bilayer *d*, area per lipid *A*, and chain order parameter *S_z_* are summarized in Table 2.

**Table 2:** **Characteristic parameters of lipid bilayers**.

Phase	$\bar{l}\;\left(\sigma_{\text{LJ}}\right)$	*d* (σ_LJ_)	$A\;\left(\sigma_{\text{LJ}}^{2}\right)$	*S_z_*
Fluid	3.3	5.48	1.68	0.42
Gel	3.8	7.64	0.96	0.72
Interdigitated	4	4.8	1.8	0.98

*$\bar{l}$ denotes the average chain length, *d* thickness of the bilayer, *A* area per lipid, and *S_z_* chain order parameter*.

Simulations were performed under constant temperature and pressure condition (NPT ensemble) for lipid bilayers with different sizes. To investigate the power spectrum of the surfaces height fluctuations, we simulated a bilayer whose upper and lower leaflets consist of 64 × 64 lipid molecules. About 17000–72000 beads were chosen for the solvent model (precise number depends on simulated phase). For performing the analysis of the Poisson’s ratio, we equilibrated rectangular bilayers consist of 12 × 24 lipid molecules per leaflet to three different phases.

**Figure 3:**
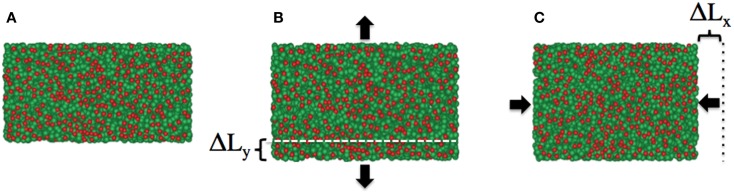
**Snapshots of the lipid membrane in fluid phase as (A) initial configuration, (B) after increasing the bilayer size in *y*-direction by a factor of (1 + η), and (C) the relaxed bilayer fixing the membrane size in *y*-direction and variable *x*-direction**. As a consequence of positive Poisson’s ratio for the bilayer in this phase, the membrane size in *x*-direction is reduced and equilibrated after a few number of MC steps. Lipid’s head beads and tail bead are shown in red and green, respectively.

To determine both *E* and ν, we need to determine one of these elastic constants separately. Utilizing the periodic boundary conditions, we introduce a method to compute the Poisson’s ratio for the surface (Abedpour et al., 2010). The Poisson’s ratio is the negative ratio of the transverse strain changes divided by the axial strain changes in a body when it is stretched or compressed along the axial direction under the tension below the proportional limit. For the infinitesimal diagonal strains, the Poisson’s ratio can be replaced by the ratio of the relative length changes as *ν_ij_* = −ΔL*_i_*/η*_j_*L*_i_*, where η*_j_* ≡ ΔL_j_/L*_j_* is defined as the fraction of the axial length change. Here *i* ≠ *j* and *i* = *x, y*, and *z*. In the method, we present here the length between neighboring lipids is rescaled by a factor of (1 + η) in axial direction, let say *y*-direction, and the subsequent change of the simulation box size in perpendicular directions, in this case *x*- and *z*-direction, are monitored. While keeping the rescaled box length (1 + η) L*_y_* constant, for fixing the pressure in the simulations, the box dimensions are now allowed to fluctuate in only the *x*- and *z*-directions. When the initial mean lengths in *x* and *z*-direction were L*_x_* and L*_z_*, new mean values of L*_x_* + ΔL*_x_* and L*_z_* + ΔL*_z_* are reached after rescaling, by re-equilibrating the system for a few number of MC steps, see Figure 3.

During the simulation, to accelerate the thermalization procedure after extending the box along the axial direction, we slightly increased the temperature in the system, which results in producing the more mobility for the particles. For the fluid phase, we set the initial temperature to *T* = 1.3 and then changed it to *T* = 1.4 for about extra 400 steps and then switched back to the original value. Similarly, for the gel and interdigitated phases, the initial temperatures were *T* = 1.08 and *T* = 1.16 and were switched to *T* = 1.2 and *T* = 1.3, respectively.

A common analysis of the elastic properties of a membrane relies on the Helfrich Hamiltonian (Helfrich, 1973), which describes the cost of elastic free enthalpy associated with fluctuations of the membrane height (deviations from flat surface). When parameterizing the membrane in Cartesian coordinates (*x,y*) →(*x,y,h*(*x,y*)) (Monge gauge), the Helfrich Hamiltonian is, for small fluctuations, given by

(4) $$H=\int \text{d}x\;\text{d}y\left[\frac{k_{\text{c}}}{2}{\left(\nabla^{2}h\right)}^{2}+\frac{\sigma }{2}{\left(\nabla h\right)}^{2}\right]$$

where *k_c_* is the bending rigidity and σ is the surface tension. Equation (4) is applied when the membrane is considered as a body with zero thickness. A generalized elastic theory for membranes with finite thicknesses was suggested by Brannigan and Brown (2006) and applied to the Lenz–Schmid model recently (West et al., 2009; Neder et al., 2010).

To determine *h*(*x, y*) from the simulations, we discretized the (*x, y*)-plane into a regular grid with spacing 2σ_LJ_, determined in each cell (*i, j*) the mean *z*-coordinates *z*_+_(*i, j*) and *z*_−_(*i, j*) of the head beads in the upper and lower leaflet, respectively, and calculated the height *h*(*i, j*) − [*z*_+_(*i, j*) + *z*_−_(*i, j*)]/2 (the average $\bar{h}$ was subtracted subsequently). For a membrane of lateral size L × L, Eq. (4) predicts:

(5) $$\left\langle {\left|ĥ\left(q\right)\right|}^{2}\right\rangle =\frac{{\text{k}}_{\text{B}}\text{T}{\text{L}}^{2}}{k_{\text{c}}q^{4}+\sigma q^{2}}$$

for the power spectrum of the fluctuations, where *ĥ*(**q**) is the Fourier transform of *h*(*x, y*) at wave vector **q**, q = |q|, and <… > denotes an equilibrium average. In the numerics, *ĥ*(**q**) is calculated from a discrete Fourier transform of *h*(*i, j*). It is clear that Eq. (5) can hold true only for a *q*-range 2π/L ≪ *q* ≪ 2π/σ_LJ_, where neither the finite system size nor the (atomistic) bead size affects the fluctuations. The corresponding *q*-range for system sizes amenable within reasonable computing time is unfortunately not large, but fits of < |*ĥ*(**q**)|^2^ > ∕k_B_TL^2^ as function of *q*^2^ in a range 0.5 ≤ *q*^2^ ≤ 1 yield a good agreement with Eq. (5) for our sizes.

## Results and Discussions

**Figure 4:**
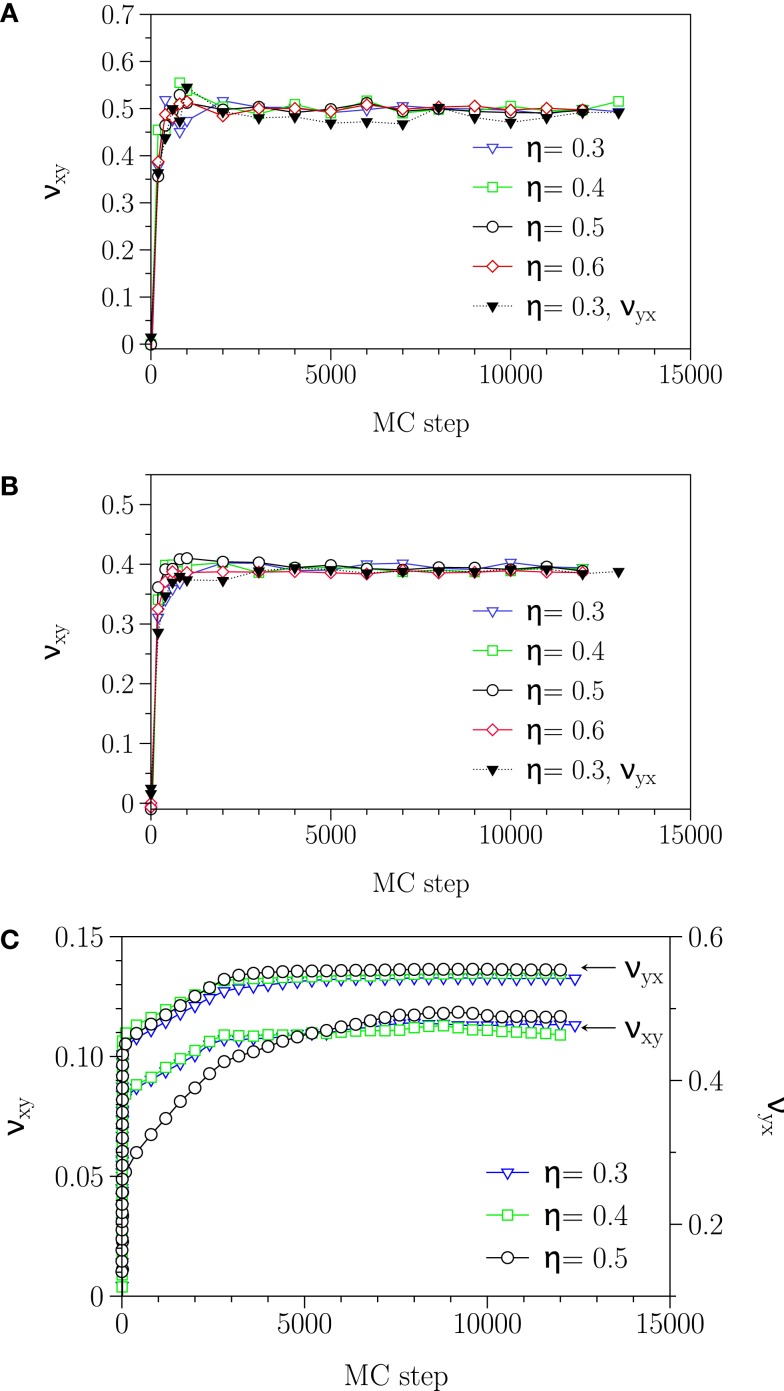
**Relaxation of −ΔL*_i_*/η*_j_*L*_i_* in (A) fluid, (B) interdigitated, and (C) gel phase**. The plateau value reached after about 5000 MC steps, independent of the value of η, yields the Poisson’s ratio ν. In the gel phase, Poisson’s ratio has different values for ν*_xy_* and ν*_yx_* and this phase acting as an anisotropic surface.

In Table 3, the obtained Poisson’s ratios ν*_yx_*, ν*_zx_*, and ν*_xy_* as well as ν*_zy_* for different phases are summarized. As demonstrated in Figure 4, the Poisson’s ratio obtained in this way is independent of the rescaling factor η as long as η is neither too large, which leads to destroy the membrane structure nor too small, which dose not produce enough free space for particles to move. According to the Table 3 and Figure 4, fluid and interdigitated phases have the same measured Poisson’s ratio for both *x* and *y*-directions. It means that these two phases are isotropic in the plane of bilayer. However, this is not the case any more for the gel phase. The measurements show that, a bilayer in the gel phase behaves as an anisotropic material, which has two distinguishably different values for the two different directions in the plane of the bilayer.

**Table 3:** **Elastic constants obtained from simulations of lipid membranes in different phases**.

Phase	*k_c_*(ϵ)	ν*_xy_*	ν*_zy_*	ν*_yx_*	ν*_zx_*	*E*(ϵ/σ^3^)
Gel	10.56	0.11	0.44	0.54	0.04	–
Fluid	5.2	0.50	0.25	0.50	0.26	0.28
Interdigitated	7.6	0.40	0.12	0.39	0.13	0.67

Conversely, when the bilayer was extended in the perpendicular direction to the tilt plane, lipids reorganize themselves in such a way that the bilayer laterally shrank. For the present work, lipids bond lengths in the *z*-direction (perpendicular to the bilayer plane) have not been rescaled. The reason is that, to observe the Poisson’s effect, the length between the beads should be rescaled by a factor, which produces enough space for particles to rearrange. However, in the *z*-direction, this increase should occur between the bonded beads inside a lipid, which causes a bond breaking. The reported values for ν*_zx_* and ν*_zy_* are the resulted relative length changes in the bilayer mean length due to the lateral extension of bilayers in *x*- and *y*-direction, respectively.

**Figure 5:**
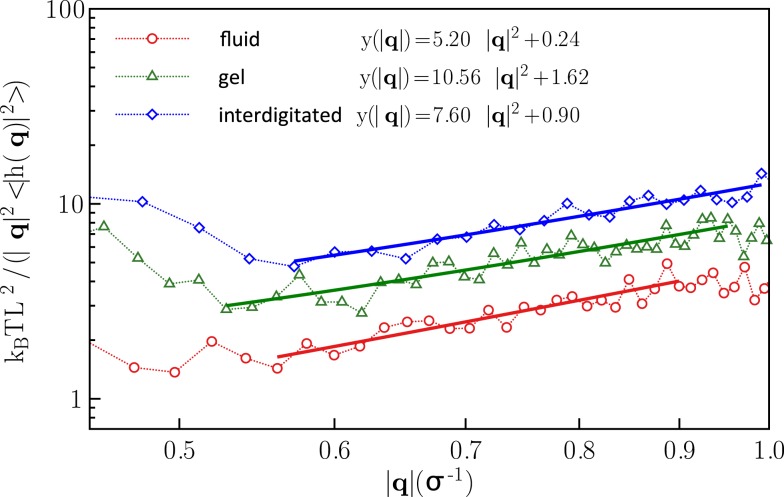
**Fluctuation spectra for the fluid, gel, and interdigitated phases and fits to the Eq. (5)**.

The values obtained for the bending rigidity *k_c_* are 5.2ϵ for the fluid phase, and 7.6ϵ for the interdigitated phase. For the gel and fluid phases, the results agree with the previous computational reports (West, 2008; West et al., 2009) and experimental findings (Falcioni et al., 1997; Liu and Zhang, 2009). The spectral density for the interdigitated phase was calculated according to the same method. We report here bending rigidity for interdigitated phase. Figure 5 illustrates the fluctuation spectra of the height for three studied phases and fits to the Eq. (5).

Within two-dimensional elasticity theory, the bending rigidity *k_c_* is related to the Poisson’s ratio *ν* and the Young’s modulus *E* according to (Landau et al., 1986)

(6) $$k_{\text{c}}=\frac{Ed^{3}}{12\left(1-\nu^{2}\right)}$$

where *d* is the mean membrane thickness, for which we obtain, 5.48σ_LJ_, and 4.86σ_LJ_ for the fluid, and interdigitated phases, respectively. Substituting the founded values for the Poisson’s ratios and the bending rigidities for bilayers in two isotropic phases into Eq. (6), one can obtain the according Young’s moduli. The Young’s moduli calculated in this way are 0.28 and 0.67 in units of ϵ/σ^3^ for the fluid and interdigitated states, respectively. Obviously, the observed anisotropicity in the gel phase dose not allow to use the above theorem for the bilayer in this phase.

The approach presented in this article could be applied to membranes with more complex lipid compositions, given that the experimentally verified interaction models exist for those lipids. The approach could also be combined with more accurate simulation of bilayers. Full atomistic simulations and in particular *ab initio* simulations, could in principle provide more accurate descriptions of the system but comes with a huge computational burden (Mashaghi et al., 2012, 2013a).

## Conclusion

We have performed Monte Carlo simulations of the coarse grained lipid bilayer model to gain insight into the mechanical properties of planar lipid membranes. By using a rescaling method, we could determine the Poisson’s ratio ν for different phases, in addition to the bending rigidity determined from an analysis of the membrane height fluctuations based on the Helfrich Hamiltonian. This allows us to calculate also the Young’s modulus *E* for different phases. The approach is accurate, easy to implement and may be applied to other membranes such as graphene (Abedpour et al., 2010), in order to predict the temperature dependence of its Poisson’s ratio and Young’s modulus. Other interesting systems to study are crystalline metallic nanowires where elastic modulus controls their structural performance and functional behavior such as their resonance frequency under oscillatory load typically applied during actuation and sensing (Chen et al., 2006; McDowell et al., 2008).

## Conflict of Interest Statement

The authors declare that the research was conducted in the absence of any commercial or financial relationships that could be construed as a potential conflict of interest.

## References

[B1] Abedpour N. Asgari R. Tabar M. R. R. (2010). Irreversibility in response to forces acting on graphene sheets. Phys. Rev. Lett. 104, 196804 10.1103/PhysRevLett.104.196804 20866989

[B2] Ayton G. Smondyrev A. Bardenhagen S. McMurtry P. Voth G. (2002). Calculating the bulk modulus for a lipid bilayer with nonequilibrium molecular dynamics simulation. Biophys. J. 82, 1226–1238 10.1016/S0006-3495(02)75479-9 11867440 PMC1301926

[B3] Brannigan G. Brown F. L. H. (2006). A consistent model for thermal fluctuations and protein induced deformations in lipid bilayers. Biophys. J. 90, 1501 10.1529/biophysj.105.075838 16326916 PMC1367303

[B4] Chen C. Shi Y. Zhang Y. Zhu J. Yan Y. (2006). Size dependence of Young’s modulus in ZnO nanowires. Phys. Rev. Lett. 96, 075505 10.1103/PhysRevLett.96.075505 16606107

[B5] Chernomordik L. V. Kozlov M. M. (2008). Mechanics of membrane fusion. Nat. Struct. Mol. Biol. 15, 675–683 10.1038/nsmb.1455 18596814 PMC2548310

[B6] Claesson M. Frost R. Svedhem S. Andersson M. (2011). Pore spanning lipid bilayers on mesoporous silica having varying pore size. Langmuir 27, 8974–8982 10.1021/la201411b 21650458

[B7] Falcioni M. Bowick M. J. Guitter E. Thorleifsson G. (1997). The Poisson ratio of crystalline surfaces. Europhys. Lett. 38, 67–72 10.1016/j.jhazmat.2010.12.101 21237558

[B8] Goetz R. Lipowsky R. (1998). Computer simulations of bilayer membranes: self-assembly and interfacial tension. J. Chem. Phys. 108, 7397–7409 10.1063/1.476160

[B9] Grest G. S. Kremer K. (1986). Molecular dynamics simulation for polymers in the presence of a heat bath. Phys. Rev. A 33, 3628–3631 10.1103/PhysRevA.33.3628 9897103

[B10] Hall L. Coluci V. Galvao D. Kozlov M. Zhang M. Dantas S. (2008). Sign change of Poisson’s ratio for carbon nanotube sheets. Science 320, 504–507 10.1126/science.1149815 18440923

[B11] Helfrich W. (1973). Elastic properties of lipid bilayers-theory and possible experiments. Z. Naturforsch. C 28, 693 4273690 10.1515/znc-1973-11-1209

[B12] Illya G. Lipowsky R. Shillcock J. C. (2005). Effect of chain length and asymmetry on material properties of bilayer membranes. J. Chem. Phys. 122, 1–6 10.1063/1.1917794 16035810

[B13] Jeon J. Voth G. (2005). The dynamic stress responses to area change in planar lipid bilayer membranes. Biophys. J. 88, 1104–1119 10.1529/biophysj.104.052183 15542558 PMC1305116

[B14] Landau L. D. Pitaevskii L. P. Lifshitz E. M. Kosevich A. M. (1986). Theory of Elasticity, Volume 7, 3rd Edn Oxford: Butterworth-Heinemann

[B15] Lenz O. (2007). Computer Simulation of Lipid Bilayers. Ph.D. thesis, University of Bielefeld, Bielefeld

[B16] Lenz O. Schmid F. (2005). A simple computer model for liquid lipid bilayers. J. Mol. Liq. 117, 147–152 10.1016/j.molliq.2004.08.008

[B17] Lindahl E. Edholm O. (2001). Molecular dynamics simulation of NMR relaxation rates and slow dynamics in lipid bilayers. J. Chem. Phys. 115, 4938–4950 10.1529/biophysj.107.121806 18192349 PMC2275712

[B18] Liu P. Zhang Y. W. (2009). Temperature-dependent bending rigidity of graphene. Appl. Phys. Lett. 94, 231912 10.1063/1.3155197

[B19] Martins B. Galvao D. (2010). Curved graphene nanoribbons: structure and dynamics of carbon nanobelts. Nanotechnology 21, 075710 10.1088/0957-4484/21/7/075710 20090201

[B20] Martins P. Malhaire C. Brida S. Barbier D. (2009). On the determination of Poisson’s ratio of stressed monolayer and bilayer submicron thick films. Microsyst. Technol. 15, 1343–1348 10.1007/s00542-009-0822-5

[B21] Mashaghi A. Partovi-Azar P. Jadidi T. Nafari N. Esfarjani K. Maass P. (2012). Interfacial water facilitates energy transfer by inducing extended vibrations in membrane lipids. J. Phys. Chem. B 116, 6455–6460 10.1021/jp302478a 22594454

[B22] Mashaghi A. Partovi-Azar P. Jadidi T. Anvari M. Panahian J. S. Nafari N. (2013a). Enhanced autoionization of water at phospholipid interfaces. J. Phys. Chem. C 117, 510–514 10.1021/jp3119617

[B23] Mashaghi S. Jadidi T. Koenderink G. Mashaghi A. (2013b). Lipid nanotechnology. Int. J. Mol. Sci. 14, 4242–4282 10.3390/ijms14024242 23429269 PMC3588097

[B24] McDowell M. Leach A. Gall K. (2008). On the elastic modulus of metallic nanowires. Nano Lett. 8, 3613–3618 10.1021/nl801526c 18947212

[B25] Mitchell J. S. Zorman C. A. Kicher T. Roy S. Mehregany M. (2003). Examination of bulge test for determining residual stress, Young’s modulus, and Poisson’s ratio of 3c-sic thin films. J. Aerosp. Eng. 16, 46–54 10.1061/(ASCE)0893-1321(2003)16:2(46)

[B26] Morone N. Fujiwara T. Murase K. Kasai R. S. Ike H. Yuasa S. (2006). Three-dimensional reconstruction of the membrane skeleton at the plasma membrane interface by electron tomography. J. Cell Biol. 174, 851–862 10.1083/jcb.200606007 16954349 PMC2064339

[B27] Nagle J. F. Tristram-Nagle S. (2000). Structure of lipid bilayers. Biochim. Biophys. Acta 1469, 159–195 10.1016/S0304-4157(00)00016-2 11063882 PMC2747654

[B28] Neder J. West B. Nielaba P. Schmid F. (2010). Coarse-grained simulations of membranes under tension. J. Chem. Phys. 132, 115101 10.1063/1.3352583 20331316

[B29] Popescu G. Ikeda T. Goda K. Best-Popescu C. A. Laposata M. Manley S. (2006). Optical measurement of cell membrane tension. Phys. Rev. Lett. 97, 218101 10.1103/PhysRevLett.97.218101 17155774

[B30] Sansom D. M. S. P. Biggin P. C. (2010). Molecular Simulations and Biomembranes: From Biophysics to Function, 1st Edn Cambridge, UK: Royal Society of Chemistry

[B31] Schmidt D. MacKinnon R. (2008). Voltage-dependent k+ channel gating and voltage sensor toxin sensitivity depend on the mechanical state of the lipid membrane. Proc. Natl. Acad. Sci. U.S.A. 105, 19276–19281 10.1073/pnas.0810187105 19050073 PMC2614752

[B32] Seto H. Yamada N. Nagao M. Hishida M. Takeda T. (2008). Bending modulus of lipid bilayers in a liquid-crystalline phase including an anomalous swelling regime estimated by neutron spin echo experiments. Eur. Phys. J. E Soft Matter 26, 217–223 10.1140/epje/i2007-10315-0 18446269

[B33] Thakkar F. M. Maiti P. Kumarana V. Ayappa K. (2011). Verifying scalings for bending rigidity of bilayer membranes using mesoscale models. Soft Matter 7, 3963–3966 10.1039/c0sm00876a

[B34] Tierney K. J. Block D. E. Longo M. L. (2005). Elasticity and phase behavior of DPPC membrane modulated by cholesterol, ergosterol and ethanol. Biophys. J. 89, 2481–2493 10.1529/biophysj.104.057943 16055540 PMC1366747

[B35] West B. (2008). Lipid-Protein Interactions in Lipid Membranes. Ph.D. thesis, University of Bielefeld, Bielefeld

[B36] West B. Brown F. L. H. Schmid F. (2009). Membrane-protein interactions in a generic coarse-grained model for lipid bilayers. Biophys. J. 96, 101 10.1529/biophysj.108.138677 18835907 PMC2710048

